# Remote Dose-Dependent Effects of Dry Needling at Distant Myofascial Trigger Spots of Rabbit Skeletal Muscles on Reduction of Substance P Levels of Proximal Muscle and Spinal Cords

**DOI:** 10.1155/2014/982121

**Published:** 2014-09-03

**Authors:** Yueh-Ling Hsieh, Chen-Chia Yang, Szu-Yu Liu, Li-Wei Chou, Chang-Zern Hong

**Affiliations:** ^1^Department of Physical Therapy, Graduate Institute of Rehabilitation Science, China Medical University, Taichung 40402, Taiwan; ^2^Department of Physical Medicine and Rehabilitation, Cheng Ching General Hospital, Taichung 40407, Taiwan; ^3^School of Chinese Medicine, College of Chinese Medicine, China Medical University, Taichung 40402, Taiwan; ^4^Department of Physical Medicine and Rehabilitation, China Medical University Hospital, Taichung 40402, Taiwan; ^5^Research Center for Chinese Medicine and Acupuncture, China Medical University, Taichung 40402, Taiwan; ^6^Department of Physical Therapy, Hungkuang University, Taichung 43302, Taiwan

## Abstract

*Background*. Dry needling at distant myofascial trigger points is an effective pain management in patients with myofascial pain. However, the biochemical effects of remote dry needling are not well understood. This study evaluates the remote effects of dry needling with different dosages on the expressions of substance P (SP) in the proximal muscle, spinal dorsal horns of rabbits. *Methods*. Male New Zealand rabbits (2.5–3.0 kg) received dry needling at myofascial trigger spots of a gastrocnemius (distant muscle) in one (1D) or five sessions (5D). Bilateral biceps femoris (proximal muscles) and superficial laminaes of L5-S2, T2-T5, and C2-C5 were sampled immediately and 5 days after dry needling to determine the levels of SP using immunohistochemistry and western blot. *Results*. Immediately after dry needling for 1D and 5D, the expressions of SP were significantly decreased in ipsilateral biceps femoris and bilateral spinal superficial laminaes (*P* < .05). Five days after dry needling, these reduced immunoactivities of SP were found only in animals receiving 5D dry needling (*P* < .05). *Conclusions*. This remote effect of dry needling involves the reduction of SP levels in proximal muscle and spinal superficial laminaes, which may be closely associated with the control of myofascial pain.

## 1. Introduction

Myofascial trigger points (MTrPs), a source of musculoskeletal pain, has been defined as a hyperirritable (hypersensitive) spot in a taut band of skeletal muscle fibers and may play a key role in the pathophysiology of myofascial pain syndrome [1].

Dry needling targeting directly the primary MTrP, if performed appropriately, is one of the effective therapies for inactivating MTrPs and alleviating pain [2–10]. However, repetitive and intensive needling manipulation may cause excess damage and increase inflammatory nociception in skeletal muscle fibers [11]. Therefore, acupuncture-like needling at a region some distance away from the painful MTrPs can provide an alternative approach to remote pain relief [12–16]. In an electrophysiological study investigating the neural mechanism of remote effects of dry needling, it has found that these effects are mediated via intact afferent neural pathways from the stimulated site to the spinal cord segments of the proximally responded affected muscle [17] and may also involve the possible effects from extrasegments of the spinal cord, such as descending pain inhibitory systems [17]. But the biochemical mechanisms underlying the nociception transmission modulated by remote effects of dry needling are still unclear.

An animal model for MTrP study on rabbit was established by Hong and Torigoe [18]. A certain hyperirritable spot (myofascial trigger spot, MTrS) in the rabbit biceps femoris muscle is similar to that in human MTrP. In this spot, local twitch responses can be elicited when the needle tip encountered a sensitive locus. As in human MTrP, spontaneous electrical activity including endplate noise (EPN) and endplate spike can also be frequently recorded within this sensitive spot [19, 20]. This animal model had been used for many studies on myofascial pain syndrome [17, 19, 21–24].

The aim of the present study was to evaluate the remote effects of dry needling treatment on the pain-related peptide, substance P (SP), in a rabbit model of MTrSs. The analgesic mechanism of remote dry needling was further elucidated by additional studies of electrophysiological EPN recordings and measurements of SP immunolabeling expressions in this rabbit model to assess and compare the alterations of SP levels in the proximally affected muscle and to explore the corresponding neuronal circuits affected by remote dry needling. This study is also designed to further investigate the dose-dependent effect of remote dry needling treatment.

## 2. Materials and Methods

### 2.1. General Design

**Figure 1:**
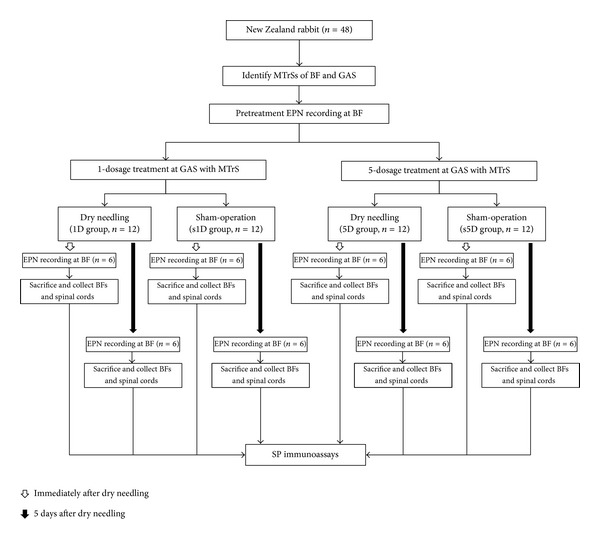
Flow chart for the animal study. Abbreviations: 1D, one-dosage dry needling; 5D, five-dosage dry needling; BF, biceps femoris; Gastrocnemius, GAS; MTrS, myofascial trigger spots, SP, substance P; s1D, sham one-dosage dry needling; and s5D, sham five-dosage dry needling.

The current experiment was designed to confirm the remote analgesic effects of dry needling at a distal muscle containing MTrSs (i.e., unilateral gastrocnemius) by quantifying the expressions of SP in the proximally affected muscles (i.e., bilateral bicep femoris muscles) and in the dorsal horns of the corresponding spinal segment (where the needling stimulation input reaches) and their suprasegments (C2-C5 and T2-T5). The MTrS of the gastrocnemius (i.e., distant muscles) on a randomly selected side was treated with predetermined dosages of dry needling. A total of 48 rabbits were randomly and equally assigned into one of the following two major groups: dry needling group (experimental) and sham-operated needling group (control). Then the animals in each group were further divided into four subgroups according to the treatment dosages at distant MTrSs of gastrocnemius. The subgroups are (1) 1D group with animals submitted to one dosage of dry needling (one session), (2) s1D group with animals submitted to one dosage of sham-operated dry needling, (3) 5D group with animals submitted to one dosage of dry needling for five consecutive days (five daily sessions), and (4) s5D group with animals submitted to one dosage of sham-operated dry needling for five consecutive days. Animals were sacrificed at two examined timepoints for SP immunoanalysis, half of the animals in each group on the day immediately after dry needling and the remaining animals on the fifth day after cessation of dry needling. The experimental design is presented in Figure 1.

### 2.2. Animal Care

The experiments were performed on adult male New Zealand rabbits (aged from 16 to 20 weeks, body weight of 2.5–3.0 kg). The animals were housed individually in standard polycarbonate tub cages lined with wood chip beddings and had free access to food and water. The cages were placed in an air-conditioned room (25 ± 1°C), with 40 dBA and a 12-hour alternating light-dark cycle (6:00 am to 6:00 pm). Each animal was housed and cared for according to the ethical guidelines of the International Association for Study of Pain in Animals [25, 26]. Effort was made to minimize discomfort and to reduce the number of animals used. All animal experiments were conducted following the procedure approved by the Animal Care and Use Committee of a university in accordance with the Guidelines for Animal Experimentation. The general experimental conditions were essentially the same as those previously described [11, 17, 27].

### 2.3. Identification and Stimulation of Myofascial Trigger Spots

Before an anesthetic is given, the most sensitive spots (i.e., MTrSs) of biceps femoris and gastrocnemius muscles were identified by finger pinch. The animal's reactions were observed (such as withdrawal of lower limb, head turning, and screaming) to confirm the exact location of an MTrS. These painful regions were marked on the skin with an indelible marker. Then the animals were anesthetized with 2% isoflurane (AErrane, Baxter Healthcare of Puerto Rico, PR, USA) in oxygen flow for induction followed by a 0.5% maintenance dose. Body temperature, monitored by a thermistor probe of a thermometer (Physiotemp Instrument, Clifton, NJ, USA) in the rectum, was maintained at approximately 37.5°C using a body temperature control system comprising a thermostatically regulated DC current heating pad and an infrared lamp. The muscle of each marked site was grasped between the fingers from behind the muscle and the muscle is palpated by gently rubbing (rolling) it between the fingers to find a taut band, which feels like a clearly delineated “rope” of muscle fibers and is roughly 10 mm in diameter. The marked sites were areas designated for dry needling treatment of gastrocnemius or electrophysiological and immunolabeling studies of biceps femoris.

### 2.4. Dry Needling of Gastrocnemius Muscle

Dry needling technique was similar to that used in our previous study [11]. For needling in MTrS of gastrocnemius, the needle (300 *μ*m in diameter and 1.5 inches in length, Yu-Kuang Industrial Co., Ltd., Taiwan) was first inserted through the skin perpendicularly at the center of the marked spot and advanced slowly and gently into the muscle. Then simultaneous needle rotation was performed to facilitate fast “in-and-out” needle movement in order to elicit as many local twitch responses as possible. For sham-operated needling, the needle was inserted into the subcutaneous layer of the marked MTrS region at a depth approximately 1-2 mm from the skin surface, without penetrating into the muscle tissues. After insertion, the needle stayed there without further movement for the same period of duration as in dry needling.

### 2.5. Recording of Endplate Noise

This procedure was performed by an investigator who was blind to the group assignment. For EPN assessment, a digital EMG machine (Neuro-EMG-Micro; Neurosoft, Ivanovo, Russia) and monopolar needle electrodes (37 mm disposable Teflon-coated model) were used. The gain was set at 20 *μ*V per division for recordings from both channels. Low-cut frequency filter was set at 100 Hz and the high-cut one was set at 1,000 Hz. Sweep speed was 10 ms per division.

The search needle was inserted into the MTrS region in a direction parallel to the muscle fibers at an angle of approximately 60° to the surface of the muscle. After initial insertion just short of the depth of the MTrS, the needle was advanced very slowly with simultaneous slow rotation to prevent it from “grabbing” and releasing the tissue suddenly to advance in a large jump. When the needle approached an active locus (EPN locus), the continuous electrical activity with amplitude higher than 10 *μ*V, that is, EPN, can be recorded. Then the needle was fixed in place to ensure that this EPN can run continuously on the recording screen with constant amplitudes for at least 3 minutes.

Five EPN recordings (25 ms each) taken before and 3 minutes after the needling treatment were randomly selected for all groups. The mean amplitude of the five random EPN recordings was analyzed and calculated for a certain measurement point for each animal.

### 2.6. Tissue Preparation

Half of the animals in each group were sacrificed on the day immediately after dry needling, and the remaining animals were sacrificed 5 days after dry needling for SP immunoassays. Animals were sacrificed under strong anesthesia by injection of saturated KCl (300 g/mL, intraperitoneal injection). Bilateral biceps femoris muscles, their corresponding segments of L5-S2, and extrasegmentsof T2-T5 and C2-C5 spinal cords were harvested. The spinal cord specimens were fixed in 4% paraformaldehyde (diluted in 0.1 mol/L PBS) and then immersed in 30% sucrose in 0.1 mol/L PBS for 2 days at 4°C for immunohistochemical staining. The muscle specimens were homogenized in T-PER tissue protein extraction reagent (Pierce Chemical Co., IL, USA) and the complete cocktail of protease inhibitors (Sigma, NY, USA) for western blot immunoassay.

### 2.7. Western Blot Analysis

Equal amounts of protein were loaded and separated in 10% Tris-Tricine SDS-PAGE gels. The resolved proteins were transferred onto polyvinylidene fluoride membranes (Millipore, Bedford, MA, USA). The membranes were blocked in 5% nonfat milk for 1 hour at room temperature and incubated overnight with primary antibodies against SP (1 : 500, Cat. # orb11399, Biorbyt, Cambridge, UK) and GAPDH (1 : 2000, ab8245, Abcam Inc. MA, USA) at a dilution of 1 : 2500 in blocking solution. The blots were then incubated with the horseradish peroxidase-conjugated goat anti-rabbit and anti-mouse IgG secondary antibody (1 : 2000, Jackson ImmunoResearch Laboratories, Inc., West Grove, PA, USA) for 1 hour at room temperature. The signals were finally visualized using an enhanced chemiluminescence detection system (Fujifilm LAS-3000 Imager, Tokyo, Japan), and the blots were exposed to X-ray. All western blot analyses were performed at least three times, and consistent results were obtained. The immunoreactive bands were analyzed using a computer-based densitometry Gel-Pro Analyzer (version 6.0, Media Cybernetics, Inc. USA). Relative intensity of western blot band for each protein was normalized to the level of GAPDH protein and presented as percentage of its sham value.

### 2.8. Immunohistochemistry for Substance P and Quantitative Analysis

The frozen spinal cord tissues were cut serially and coronally into sections of 4 *μ*m thickness by a freezing microtome. Each spinal cord specimen produced approximately 100 sections. Each staining assay was examined in 10 alternate sections per rabbit, which were selected by a systematic-random series with a random start for analysis. The sections were first mounted on poly L-lysine (Sigma, P8920)-coated slides and then blocked in 10% normal goat serum (in PBS with 0.3% Triton X-100, Jackson ImmunoResearch Laboratories, Inc., West Grove, PA, USA). Subsequently, they were incubated overnight at 4°C with rat monoclonal anti-SP antibody (1 : 200, MAB356, Millipore, CA, USA). The sections were then incubated with biotinylated goat anti-rat IgG secondary antibody (1 : 500, Jackson ImmunoResearch Laboratories, Inc., West Grove, PA, USA) for 1 hour at room temperature. After being washed, the sections were incubated with a streptavidin-horseradish peroxidase conjugate (Jackson ImmunoResearch Laboratories, Inc., West Grove, PA, USA). The sections were visualized as brown precipitates by adding 0.2 mg/mL 3,3′-diaminobenzidine (DAB, Pierce, Rockford, IL, USA) as a substrate. Negative control slides omitting either primary antibodies or secondary antibodies were also prepared for comparison.

The slides were examined and photographed at three randomly selected fields of superficial dorsal horn (laminae I-II; superficial, the localization of nociresponsive neurons) at 200x magnification using a light microscope (BX43, Olympus America Inc. NY, USA) and a cooled digital color camera with a resolution of 1360 × 1024 pixels (DP70, Olympus America Inc. NY, USA). Images were saved and adjusted to equalize contrast and brightness with Adobe Photoshop (CS3, San Jose, CA); no other modifications were made.The digital images were analyzed using a computer-based morphometry, Image-Pro Plus 4.5 software (Media Cybernetics, Silver Spring, USA). According to the automatically calculated parameters, the area labeled by DAB-stained strong-positive staining cells for SP immunoreactivity (SP-IR) was measured. The percentage of the positive and strong SP-IR pixels to total stained pixels in superficial lamina of spinal dorsal horn (%) was analyzed.

### 2.9. Statistical Analysis

All data were expressed as mean ± standard deviation (SD). The differences in SP-IR levels in animals submitted to dry needling and sham operation were calculated. One-way analysis of variance (ANOVA) was employed to determine the differences in SP-IR levels among groups. Post hoc comparisons between groups were examined using Scheffe's method. A *P* value of <.05 was considered statistically significant. All data were analyzed using SPSS version 12.0 for Windows (SPSS Inc., IL, USA).

## 3. Results

### 3.1. Changes in Amplitudes of EPN in Biceps Femoris Induced by Dry Needling at Distant MTrSs

**Figure 2:**
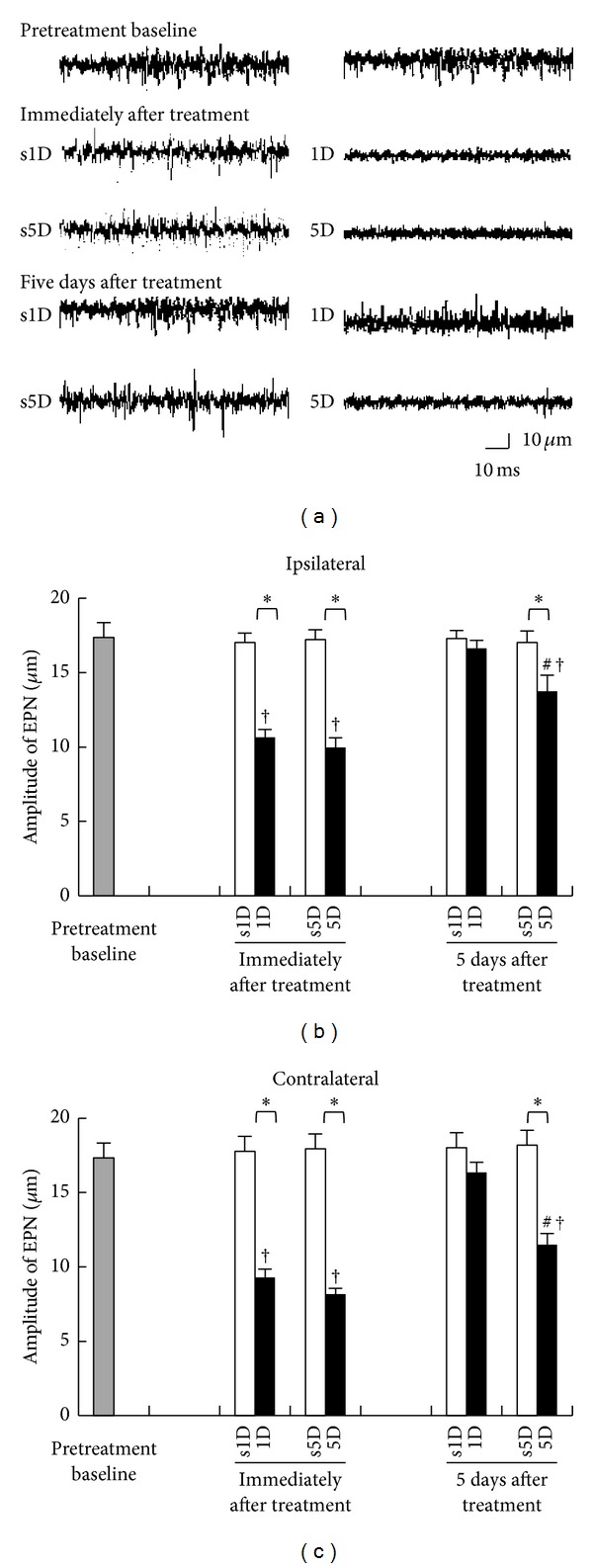
Changes in EPN amplitude measured at MTrS of biceps femoris before, immediately, and 5 days after one-(1D) and five-dosage (5D) of dry needling at gastrocnemius in experimental (1D and 5D groups) and sham-operated groups (s1D and s5D groups). Sample EPN recordings (a) in rabbits from 1D, s1D, 5D, and s5D groups. (b) Quantification of EPN amplitude in biceps femoris ipsilateral (b) and contralateral (c) to dry needling side expressed as mean ± SD. ∗ represents significant difference (*P* < .05) compared with sham-operated groups (s1D and s5D). # represents significant difference (*P* < .05) between 1D and 5D groups. †: *P* < .05 represent significant differences compared with pretreatment values.

Figure 2 shows the alterations of mean EPN amplitude recorded from biceps femoris ipsilaterally and contralaterally to the dry needling side at gastrocnemius muscle. As can be seen, there were significant differences among the four groups at any examined timepoint (ANOVA, all *P* < .05). Compared with the pretreatment levels, the EPN amplitudes of ipsilateral biceps femoris were significantly decreased immediately after dry needling treatment (*P* < .05; Figure 2(a)). However, the EPN amplitudes after sham-operated needling showed no marked changes (*P* > .05).

There were also significant differences in EPN amplitudes recorded immediately after treatment between dry needling and sham-operation groups at any dosage (1D versus s1D, 5D versus s5D, all *P* < .05; Figure 2(a)). Five days after treatment, the marked reduction in EPN amplitude was no longer observed in the 1D group (1D versus s1D, *P* > .05). However, the 5D group showed significant decrease in EPN amplitude when compared with the s5D group (5D versus s5D, *P* < .05; Figure 2(a)) and the 1D group (1D versus 5D, *P* < .05; Figure 2(b)). The EPN recordings from biceps femoris contralateral to the dry needling side presented similar trends of amplitude changes (Figure 2(c)).

### 3.2. Changes in SP-IR of Biceps Femoris Induced by Dry Needling at Distant MTrSs

**Figure 3:**
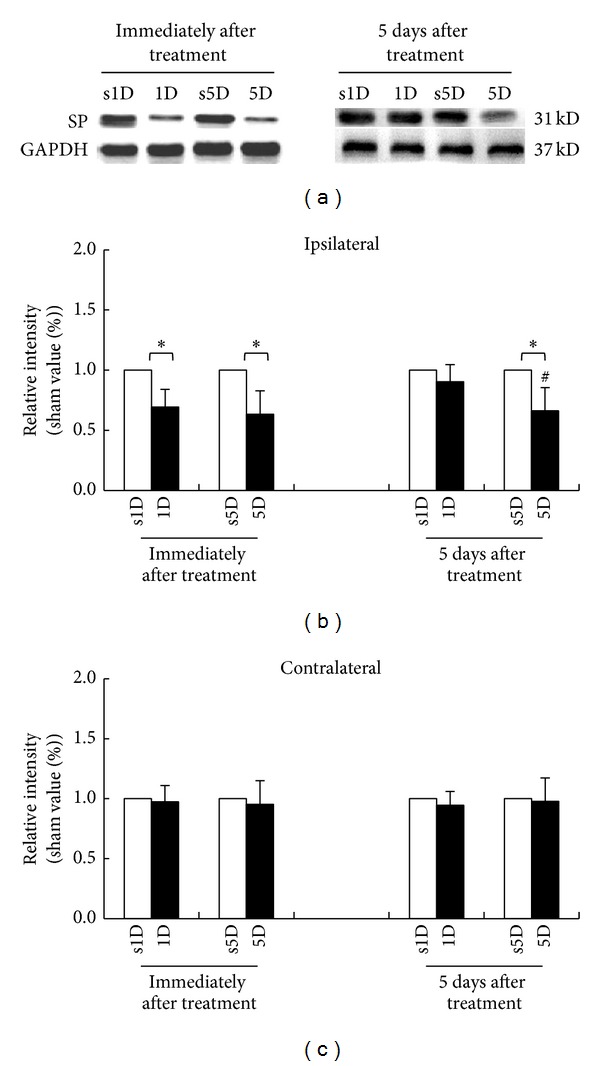
Alterations of substance P (SP) level at biceps femoris muscle after one-(1D) and five-dosage (5D) of dry needling at gastrocnemius in experimental (1D and 5D groups) and sham-operated groups (s1D and s5D groups). Representative western blot photographs (a) in ipsilateral biceps femoris immediately and 5 days after dry needling at gastrocnemius. Quantification of SP levels in biceps femoris ipsilateral (b) and contralateral (c) to dry needling side expressed as mean ± SD. ∗ represents significant difference (*P* < .05) compared with sham-operated groups (s1D and s5D). # represents significant difference (*P* < .05) between 1D and 5D groups.

Figure 3 presents the SP-IR in biceps femoris immediately and 5 days after treatments for all groups. As can be seen, there were significant differences among the four groups at any examined timepoint (ANOVA, all *P* < .05). Immediately after treatment, 1D and 5D groups showed significant decrease in SP-IR levels in biceps femoris ipsilaterally to the needling side when compared with s1D and s5D groups, respectively (Scheffe's method, 1D versus s1D, 5D versus s5D, all *P* < .05; Figures 3(a) and 3(b)).

Five days after treatment, the 5D group still showed marked decrease in SP-IR levels when compared with the s5D group (Scheffe's method, *P* < .05) but there was no difference in SP-IR levels between 1D and s1D groups (*P* > .05; Figure 3(b)). Moreover, there were no significant differences in SP-IR in biceps femoris contralaterally to the needling side between 1D and s1D, as well as 5D and s5D groups at any examined timepoint (Scheffe's method, 1D versus s1D, 5D versus s5D, all *P* > .05; Figure 3(c)).

### 3.3. Changes in SP-IR Cells of Spinal Dorsal Horns Induced by Dry Needling at Distant MTrSs

#### 3.3.1. SP Expressions in L5-S2 Segments

**Figure 4:**
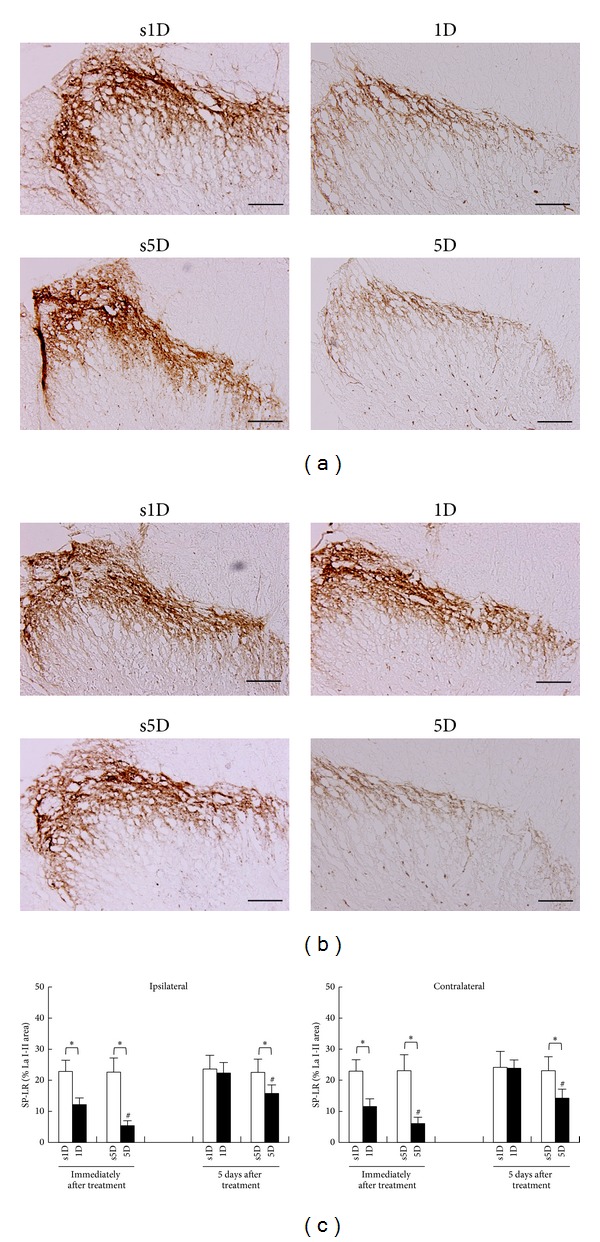
Alterations of substance P (SP) levels at superficial laminae of L5-S2 after one-(1D) and five-dosage (5D) of dry needling at gastrocnemius in experimental (1D and 5D groups) and sham-operated groups (s1D and s5D groups). The representative photomicrographs indicate immunohistochemical labeling for SP in ipsilateral dorsal horns of lumbosacral spinal cords at the timepoints immediately (a) and 5 days (b) after dry needling. Histograms indicate the quantitative analysis of SP immunoreactivity in ipsilateral and contralateral spinal cords (c). ∗ represents significant difference (*P* < .05) compared with sham-operated groups (s1D and s5D). # represents significant difference (*P* < .05) between 1D and 5D groups (scale bar = 100 *μ*m).

The SP-IR patterns in L5-S2 segments for the four groups immediately and 5 days after treatments are presented in Figures 4(a) and 4(b), respectively. Qualitative analysis of the SP-IR in superficial laminae of the L5-S2 dorsal horn showed different patterns of reactivity between dry needling and sham-operation groups (Figure 4(c)). There were significant differences among the four groups at any examined timepoint (ANOVA, all *P* < .05).

In sham-operated controls, SP-IR was expressed bilaterally in the nuclei of cells of the superficial laminae in L5-S2 dorsal horn spinal cords, which showed no statistically significant differences between s1D and s5D groups at each examined timepoint (Scheffe's method, *P* > .05; Figure 4(c)). The nuclei of SP-IR neurons were visualized as brown precipitates and there was also some cytoplasmic staining. Most of the SP-IR cells were distributed in the bilateral superficial laminae (I-II) of the dorsal horn. Immediately after treatment, 1D and 5D groups showed significant reduction in SP-IR levels in bilateral superficial laminae when compared with s1D and s5D groups, respectively (Scheffe's method, 1D versus s1D, 5D versus s5D; all *P* < .05; Figure 4(a)).

Overall, the spinal cord sections had faint SP labelings in bilateral superficial laminae of dorsal horns in both groups submitted to dry needling, as confirmed by the quantitative analysis, which also showed statistically significant differences between 1D and 5D groups (Scheffe's method, 1D versus 5D, *P* < .05; Figure 4(c)). Five days after treatment, there was no significant difference in SP-IR level between the 1D group and the s1D group (Scheffe's method, 1D versus s1D, *P* > .05; Figure 4(b)). However, the 5D group still showed significant reduction in the SP-IR level in bilateral superficial laminae when compared with the s5D group (Scheffe's method, 5D versus s5D, *P* < .05; Figure 4(b)) and the 1D group (Scheffe's method, 1D versus 5D, *P* < .05; Figure 4(c)).

#### 3.3.2. SP Expressions in C2-C5 and T2-T5 Suprasegments

**Figure 5:**
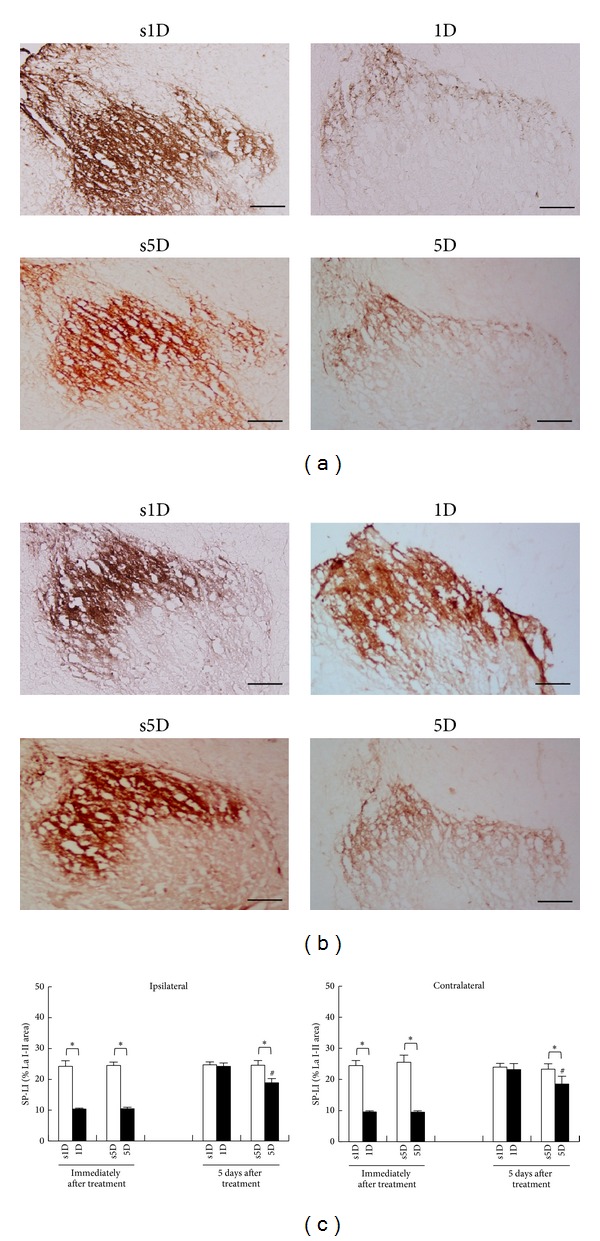
Alterations of substance P (SP) levels at superficial laminae of T2-T5 after one-(1D) and five-dosage (5D) of dry needling at gastrocnemius in experimental (1D and 5D groups) and sham-operated groups (s1D and s5D groups). The representative photomicrographs indicate immunohistochemical labeling for SP in ipsilateral dorsal horns of lumbosacral spinal cords at the timepoints immediately (a) and 5 days (b) after dry needling. Histograms indicate the quantitative analysis of SP immunoreactivity in ipsilateral and contralateral spinal cords (c). ∗ represents significant difference (*P* < .05) compared with sham-operated groups (s1D and s5D). # represents significant difference (*P* < .05) between 1D and 5D groups (scale bar = 100 *μ*m).

**Figure 6:**
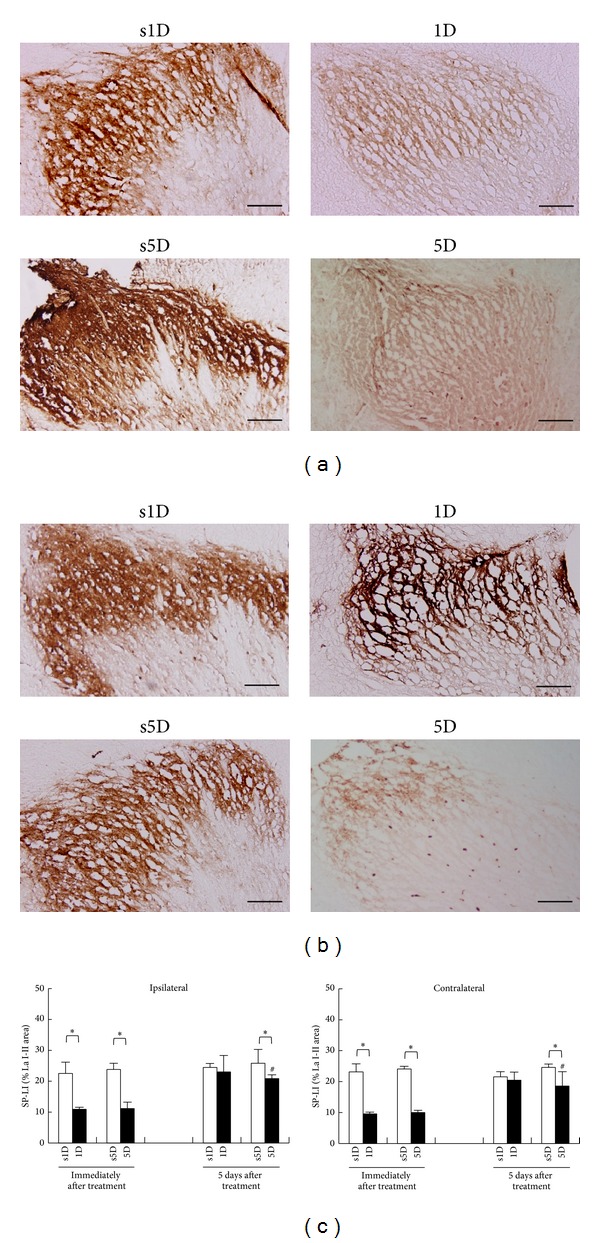
Alterations of substance P (SP) levels at superficial laminae of C2-C5 after one-(1D) and five-dosage (5D) of dry needling at gastrocnemius in experimental (1D and 5D groups) and sham-operated groups (s1D and s5D groups). The representative photomicrographs indicate immunohistochemical labeling for SP in ipsilateral dorsal horns of lumbosacral spinal cords at the timepoints immediately (a) and 5 days (b) after dry needling. Histograms indicate the quantitative analysis of SP immunoreactivity in ipsilateral and contralateral spinal cords (c). ∗ represents significant difference (*P* < .05) compared with sham-operated groups (s1D and s5D). # represents significant difference (*P* < .05) between 1D and 5D groups (scale bar = 100 *μ*m).

A similar trend of SP-IR expression at L5-S2 segments was also observed in bilateral superficial laminae at T2-T5 (Figure 5(a)) and C2-C5 levels (Figure 6(a)). There were significant differences in SP expressions among the four groups at any examined timepoint (ANOVA, all *P* < .05). Immediately after treatment, 1D and 5D groups showed statistically significant reduction in SP-IR levels in bilateral superficial laminae at T2-T5 and C2-C5 levels when compared with s1D and s5D groups, respectively (Scheffe's method, 1D versus s1D, 5D versus s5D, all *P* < .05). In contrast, there were no significant differences between 1D and 5D groups at any examined level (Scheffe's method, 1D versus 5D, T2-T5: *P* > .05; C2-C5: *P* > .05; Figures 5(c) and 6(c)).

Five days after treatments, the 1D group showed no significant change in SP-IR at T2-T5 and C2-C5 levels when compared with the s1D group (Scheffe's method, 1D versus s1D group, *P* > .05). However, the spinal sections in the 5D group showed a reduction in SP-IR expressions when compared with those in the s5D group (Scheffe's method, 5D versus s5D, *P* < .05; Figures 5(b) and 6(b)). The 5D group also showed significant reduction in SP-IR levels in bilateral superficial laminae when compared with the 1D group at each examined level (Scheffe's method, 1D versus 5D, T2-T5: *P* < .05; C2-C5: *P* < .05; Figures 5(c) and 6(c)).

#### 3.3.3. Differences in SP-IR Patterns among Spinal Cord Levels

**Figure 7:**
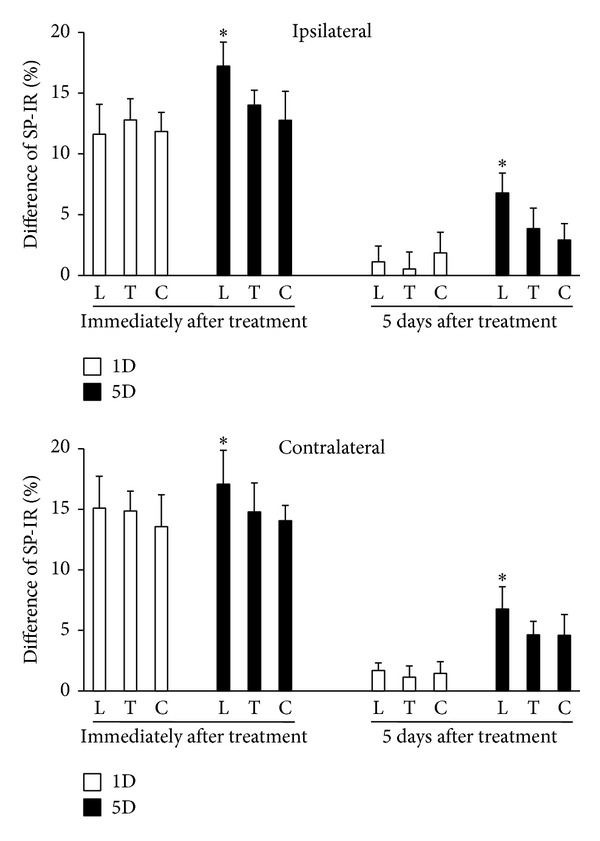
Differences in substance P (SP) levels at ipsilateral and contralateral superficial laminae of L5-S2 (L), T2-T5 (T), and C2-C5 (C) between animals after one-(1D) and five-dosage (5D) of dry needling at gastrocnemius in experimental (1D and 5D groups) and sham-operated groups (s1D and s5D groups). ∗ represents significant difference (*P* < .05) compared with SP levels of thoracic and cervical dorsal horns.

Comparison among SP-IR patterns of C2-C5, T2-T5, and L5-S2 levels shows no statistically significant differences in either 1D or s1D group at any examined timepoint (ANOVA, *P* > .05). However, the differences in SP-IR between 5D and s5D groups were significant among various spinal cord levels at all examined timepoints of either immediately or 5 days after treatments (ANOVA, *P* < .05). The reduction of SP-IR cells in the lumbosacral dorsal horn was significantly higher in comparison with that in cervical or thoracic cells (Scheffe's method, all *P* < .05, Figure 7).

## 4. Discussion

This is the first study showing that short-term and long-term dry needling at distant MTrSs can induce suppression of SP levels in the proximal muscle and spinal cord dorsal horns. The results also suggest that maintenance effects of remote dry needling may contribute to the extrasegmental desensitization effect. There is strong indication that both neural and biochemical effects are involved in the mechanisms of remote pain control.

Dry needling targeting the MTrPs for pain relief has its basis in theories similar, but not exclusive, to traditional acupuncture. The electrophysiological outcomes affected by acupuncture and dry needling are similar [17]. In our previous studies, decreases in EPN amplitude in proximal muscles containing MTrSs were found after dry needling at the MTrSs of a distant muscle when compared with pretreatment baseline EPN amplitude in animals with intact neural circuits [17]. Similar findings were found in humans receiving acupuncture at remote acupoints [15, 16]. Fernández-Carnero et al. also found an increase in spontaneous electrical activity at an MTrP region during persistent noxious stimulation at another distant MTrP, followed by suppression of electrophysiological irritability after cessation of needling [28]. The evidences reveal that dry needling at distant MTrS could decrease the irritability of the proximal MTrS by suppression of EPN in prevalence and amplitude. In the present study, the results obtained are in line with those of earlier studies, showing that EPN amplitudes were significantly reduced after a single or five dosages of dry needling when compared with those after sham operation.

SP has been widely proposed as being involved in delivering nociceptive information from the peripheral receptors to the spinal dorsal horn and supraspinal processing centers, thus leading to central sensitization [29–34]. Interventions that inhibit SP signaling pathways generally show antinociceptive effects in animal models [35, 36]. Electroacupuncture can reduce the noxious nerve stimulation-induced release of SP from both central terminals and peripheral endings of the primary sensory neurons [37]. Immunohistochemical studies showed that electroacupuncture of “Zusanli” (ST-36) could depress the pain response and inhibit spinal dorsal horn SP release [38]. Electroacupuncture can also suppress immunoreactive SP accumulation induced by tooth pulp stimulation in the superficial layers of the trigeminal nucleus caudalis [39]. Therefore, SP may be an important transmitter in mechanism of acupuncture analgesia. The present result on dry needling-induced suppression of spinal dorsal horn SP was consistent with those of previous studies described above, implying that short- and long-term remote dry needling probably produce analgesic effect. In addition, the finding of reduction in SP expression in extrasegments, dorsal horns of C2-C5, and T2-T5 after dry needling was similar to our previous results [17], which reported supraspinal control of spinal inhibitory interneurons induced by dry needling at distant MTrSs. The extrasegmental desensitization effect involving a more generalized system of analgesia is probably activated by remote dry needling.

SP is also likely to be involved in the pathogenesis of musculoskeletal pain. A recent study has also found higher SP levels in active MTrPs compared with control latent or absent MTPs [40]. Mean optical density of the immunostaining for SP was statistically greater in trapezius muscle of patients with myofascial pain syndrome when compared with specimens from patients with fibromyalgia. Extensive studies demonstrate that SP accumulated in MTrPs of skeletal muscles is associated with pain and inflammation [17, 40, 41]. The current findings of reduction in SP level in proximal muscle (biceps femoris) ipsilateral to the needling side after dry needling at a distant muscle containing MTrSs may be related to pain control.

Suppression of SP expressions was of a higher extent in animals submitted to 5D dry needling than those submitted to 1D dry needling, indicating immediate dose-dependent effects. On the other hand, five days after cessation of dry needling, the suppression effect persisted and even became more pronounced in laminae I to II at L5-S2 levels in animals submitted to 5D dry needling, demonstrating prolonged dose-dependent effects. The immediate pain relief by 1D dry needling is probably elicited by the neural effect as demonstrated by changes in EPN. However, the long-term or accumulated effect of pain relief by 5D dry needling may be accompanied by and mediated via biochemical changes in addition to neural effects. Therefore, repetitive or extensive remote needling may provide better effects on reduction in SP levels for control of myofascial pain. It is commonly accepted that electroacupuncture of higher intensities and longer pulse durations can produce persistent analgesia [42].

## 5. Conclusion

The hypothesis that dry needling at distant MTrSs could modulate irritability of proximal MTrSs by altering the EPN amplitude and SP levels of muscle and spinal cords is supported by the present results. The findings of this study might elucidate the biochemical mechanisms induced by remote effects of dry needling. Accordingly, the practice of dry needling at some distance away from the painful site may facilitate decreased MTrP sensitivity in any muscle within the same levels of spinal innervations via activating the mechanisms of segmental and extrasegmental desensitization. Further understanding of the analgesic action of dry needling in treating soft tissue pain can contribute to develop new therapeutic strategies for treating myofascial pain syndrome.
